# Elbow joint biomechanics during ADL focusing on total elbow arthroplasty - a scoping review

**DOI:** 10.1186/s12891-023-06149-8

**Published:** 2023-01-18

**Authors:** Daniëlle Meijering, Roos GA Duijn, Alessio Murgia, Alexander L. Boerboom, Denise Eygendaal, Michel PJ van den Bekerom, Sjoerd K. Bulstra, Martin Stevens, Riemer JK Vegter

**Affiliations:** 1 Department of Orthopedic Surgery, University of Groningen, University Medical Center Groningen, Groningen, The Netherlands; 2 Department of Human Movement Sciences, University of Groningen, University Medical Center Groningen, Groningen, The Netherlands; 3 Department of Orthopedics and Sports Medicine, Erasmus University Medical Center, Rotterdam, The Netherlands; 4 Department of Orthopedic Surgery, OLVG, Amsterdam, The Netherlands; 5 Department of Human Movement Sciences, Faculty of Behavioral and Movement Sciences, Vrije Universiteit Amsterdam, Amsterdam Movement Sciences, Amsterdam, The Netherlands

**Keywords:** Elbow joint loading, Elbow prosthesis, Arthroplasty, Biomechanical analysis, Activities of daily living

## Abstract

**Background:**

Overloading is hypothesized to be one of the failure mechanisms following total elbow arthroplasty (TEA). It is unclear whether the current post-operative loading instruction is compliant with reported failure mechanisms. Aim is therefore to evaluate the elbow joint load during activities of daily living (ADL) and compare these loads with reported failure limits from retrieval and finite element studies.

**Methods:**

A scoping review of studies until 23 November 2021 investigating elbow joint load during ADL were identified by searching PubMed/Medline and Web of Science. Studies were eligible when: (1) reporting on the elbow joint load in native elbows or elbows with an elbow arthroplasty in adults; (2) full-text article was available.

**Results:**

Twenty-eight studies with a total of 256 participants were included. Methodological quality was low in 3, moderate in 22 and high in 3 studies. Studies were categorized as 1) close to the body and 2) further away from the body. Tasks were then subdivided into: 1) cyclic flexion/extension, 2) push-up, 3) reaching, 4) self-care, 5) work. Mean flexion–extension joint load was 17 Nm, mean varus-valgus joint load 9 Nm, mean pronation-supination joint load 8 Nm and mean bone-on-bone contact force 337 N.

**Conclusion:**

The results of our scoping review give a first overview of the current knowledge on elbow joint loads during ADL. Surprisingly, the current literature is not sufficient to formulate a postoperative instruction for elbow joint loading, which is compliant with failure limits of the prosthesis. In addition, our current instruction does not appear to be evidence-based. Our recommendations offer a starting point to assist clinicians in providing informed decisions about post-operative instructions for their patients.

**Supplementary Information:**

The online version contains supplementary material available at 10.1186/s12891-023-06149-8.

## Background

Total elbow arthroplasty (TEA) is a viable option for patients with end-stage, symptomatic elbow pathology such as post-traumatic arthritis, post-traumatic deformities, primary osteoarthritis, and rheumatoid arthritis [1]. TEA survival rate is limited by complications (10–40% complication rates) and mechanical failures with aseptic loosening and polyethylene (PE) wear, leading to 10-year survival rates of 80–85% [2–4]. These survival rates are low compared to hip and knee arthroplasties (~ 95%) [5, 6]. Understanding the mechanisms of TEA failure may help when formulating implications for clinical practice, in order to improve implant survival rates and lower complication rates.

Based on retrieval studies, several mechanisms have been hypothesized to cause TEA failure. First, overloading of the prosthesis during activities of daily living (ADL) is thought to result in PE wear, with consequent instability of the hinge, asymmetric varus-valgus load transmission, and PE particle disease. This cascade results in bone and tissue destruction and loosening of the implant. For example, PE wear of the Coonrad Morrey (Zimmer Biomet, USA) elbow prosthesis, retrieved at revision surgery, showed asymmetrical wear with PE bushings deformed to an elliptical shape, which is mainly attributed to varus-valgus and torsional loading of the elbow [7].

Next to retrieval studies, finite element studies examining the stress distribution on the elbow prosthesis have also shed light on the failure mechanisms of TEA. Lo and Lipman [8], studying the Coonrad Morrey (Zimmer Biomet, USA) elbow prosthesis, concluded that 5 Nm varus-valgus load at the ulno-humeral joint was sufficiently high to result in stresses exceeding the theoretical yield strength of PE (ultrahigh molecular weight PE; UHMWPE). These stresses led to extrusion and non-reversible PE deformation, eventually causing wear.

In conclusion, both retrieval and prosthetic design studies report elbow load values that lead to failure and thus should not be exceeded following TEA. However, the consequences of these findings for clinical practice with patients following TEA remain unknown, since elbow loads actually experienced by patients during ADL are not well established. Daily tasks can result in high loads and thus stresses on the elbow depending on the amount of load being lifted and the movement being executed [9]. Our current clinical practice is to instruct patients to limit weight lifting to 1 kg in general and to 5 kg incidentally. Still, depending on the type of movement and how it is executed, similar weights can lead to different loads on the elbow [9]. Moreover, not all tasks involve external weight yet still require load on the elbow, such as rising from a chair or steering a car. Therefore, in the current review we aim to investigate the literature on reported elbow loads during different ADL tasks. It is currently unclear whether elbow loads experienced during ADL tasks exceed the reported failure limits of the prosthesis. It is also unclear whether the experienced loads and failure limits relate to our postoperative instruction. The overview of elbow loads during ADL tasks is expected to create a basis for better clinical practice and guide more informed decisions on which tasks should be avoided following TEA.

Hence, the main research question of the current review is: What is the elbow joint load (bone-on-bone contact force and net joint torque) during different ADL tasks, and do these loads exceed the failure limits as reported in retrieval and finite element studies on TEA?

## Methods

The Preferred Reporting Items for Systematic Reviews and Meta-Analyses extension for Scoping Reviews (PRISMA-ScR) guidelines were followed. The review was registered in an international prospective register of scoping reviews ‘Science Framework’. The protocol is registered online and can be accessed electronically at: https://osf.io/823vt/

### Literature search and study selection

With the assistance of a clinical librarian, a systematic literature search was performed on 23 November 2021 in two online databases (PubMed/Medline and Web of Science). The following terms were used: [Elbow], [Elbow Joint], [Arthroplasty], [TEA], [Biomechanical]. The search was performed using the filters “Dutch” and “English”. Full search details are available in Additional file 1  Appendix 1.

Identified articles were imported to Endnote (Philadelphia, USA). Duplicates were removed. Based on title and abstract, two independent reviewers (DM and RGAD) identified potentially relevant articles for review of the full text. In case of disagreement, a third author was consulted (AM). The reference list of the included articles was manually checked to avoid missing relevant articles. The authors independently selected articles. Studies were not blinded for author, affiliation or source.

### Eligibility criteria

Studies were eligible when: (1) reporting on the elbow joint load in native elbows or elbows with an elbow arthroplasty in adults; (2) full text article was available. A study was excluded if it only contained specific sport analysis. Studies in patients with neurological comorbidities (i.e. cerebral palsy, stroke, spinal cord injury) were excluded. Animal studies and cadaveric studies were also excluded.

### Data extraction

After initial selection, data from eligible studies were extracted based on a predefined extraction template. The following data and baseline parameters were recorded when available: author and publication year, number of participants, participant characteristics (sex, age, indication for TEA, type of TEA, radial head status, ligament status (if applicable)), and methods (tracking system, ADL tasks). Primary objective was to report on elbow joint load along the local axes (flexion, extension, varus, valgus, pronation, supination). For all axes the largest measured load (peak load) per task was taken. Last, load definitions were extracted using the following categories of net joint torque (Nm), interaction torque (Nm), and bone-on-bone contact force (N). In order to be able to compare reported elbow loads between studies, load definitions were extracted based on the ISB recommendations [10].

**Fig. 1:**
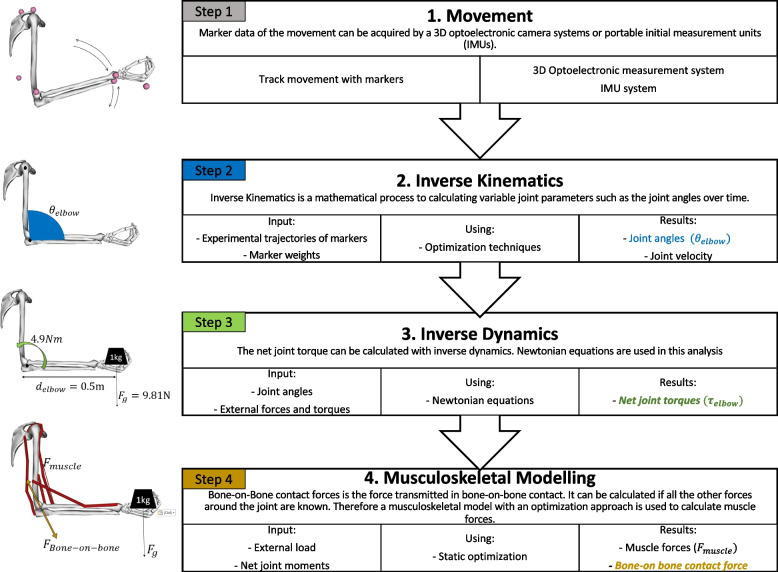
Steps to analyze joint torque. θ_*elbow*_ = elbow joint angle, ${\tau }_{elbow}$ = net joint torque, ${d}_{elbow}$= moment arm (distance between force vector and rotation point (the elbow axis), ${F}_{g}$ = gravitational force ($m*g$) and ${F}_{Bone-on-bone}$ = internal bone-on-bone contact force

Figure 1 describes four steps of increasing detail in the study of joint torque, as defined by the ISB recommendations [10]. To be able to compare elbow loads between studies, methods were screened to check whether they used the following steps. First, movement can be tracked using markers. Marker data of the movement can be acquired by using a 3D optoelectrical camera system or portable inertial measurement unit (IMU) (step 1). Next, a mathematical process called inverse kinematics is used to calculate joint parameters (joint angles and joint velocity), by using marker trajectory data from step 1 (step 2). The net joint torque can be calculated using inverse dynamics. The inverse dynamics method estimates the torques and forces needed to generate a motion. Position data of the segments (steps 1 and 2) are put into a biomechanical model (step 3). The net joint torque can then be calculated by the formula ( $${\tau }_{elbow}={d}_{elbow}*{F}_{g}).$$ A larger moment arm ($${d}_{elbow})$$ or a larger gravitational force ($${F}_{g})$$ results in a higher net joint torque ($${\tau }_{elbow}$$). A 1 kg mass in the hand leads to an elbow moment depending on the moment arm (i.e. the type of movement). Translating a mass to a moment is therefore difficult. Interaction torque only occurs by multi-joint movements, for example by reaching where both elbow and shoulder joints are active. Generation of the resulted joint-torque is complicated by the presence of interaction torque. The interaction torque is due to initial torque, centripetal torque, and Coriolis torque [11]. Bone-on-bone contact force is the force transmitted in bone-on-bone contact [12], and can be calculated if all the other forces (i.e. muscle, external, gravitational) around the joint are known. An optimization process thus needs to be performed to calculate the muscle force, which can be done using a musculoskeletal model (step 4) [13].

### Methodological quality assessment

The methodological quality of included studies was evaluated using a checklist by Heyward et al. [14] (Additional file 1 Appendix 2). For each question a score of 1 was given for an ‘adequate’ or ‘yes’ response, 0.5 for a ‘partial’ or ‘limited’ response, and 0 was awarded for a ‘no’, ‘not stated’ or ‘inadequate’ response. A maximum score of 8 was possible. Studies were considered low quality if they scored 0–3.5 points, moderate quality 4–5.5 points, and high quality 6–8 points. These ranges were chosen arbitrarily. Methodological quality assessment was assigned by two authors, any differences in scoring were resolved by consensus (DM and RGAD).

## Results

### Selection of literature

**Fig. 2:**
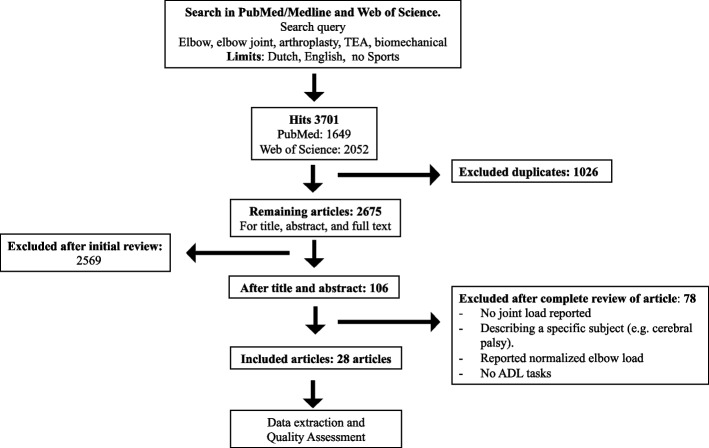
Flow-chart

An initial search yielded 3701 potentially relevant studies. After removal of duplicates, 2675 articles were identified. After evaluation of titles and abstracts, the remaining 106 papers were retrieved for detailed assessment of the full-text manuscript. Seventy-eight studies were excluded since they did not report on elbow joint load, so a total of 28 articles [9, 15–41] were included (Fig. 2).

### Quality assessment

Of the articles included, three studies [16, 30, 32] were of low quality, 22 [9, 15, 18–27, 29, 31, 33–39] of moderate quality and three [17, 28, 40] of high quality. Areas of improvement for most studies were description of inclusion and exclusion criteria, and of validity and reliability of measurement tools. Details of these are shown in Additional file 1 Appendix 3.

### Study characteristics

Overall, a total of 256 participants (203 male: 53 female) were included. Age ranged from 17 to 59 years (30 ± 11). The number of participants per study ranged from 1 – 30. Table 1 presents an overview of the study characteristics.

**Table 1:** Overview of study characteristics

First Author	Year	Number of participants (n)	Male (n)	Age (Mean)	Weight (kg)	Close to body/Further away	Type of task	Sort torque	Peak F/E **load** (Nm)	Peak V/V l**oad** (Nm)	Peak Pro/Sup **load** (Nm)	External weight applied (kg)	Bone-bone contact force (N)
Almeida [15]	1995	4	4	32		Further	C	Net	40				
An [16]	1992	9	9			Further	P	Net	23				304
Balendra [17]	2017	10	10	24	81	Further	R	Net	8	3	1	0.45	
									15	7	2	0.9	
Ballaz [18]	2016	12	6	23		Close	C	Net	2			0	
									5			1	
									9			2	
									11			3	
Beer [19]	2004	5	3	59		Further	R	Net	15				
Challis [20]	1994	1	1		65	Close	C	Net	46				
Chou [21]	2001	11	11	26	69	Further	P	Net	22	11	8		353
Chou [22]	2002	8	8	17	69	Further	P	Net	51	20	17		441
Chou [25]	2008	10	10	27	63	Further	P	Net	16	10			304
Chou [23]	2009	15	15	23	68	Further	P	Net					422
Chou/Hsu [26]	2011	14	14	25	66	Further	P	Net	28	7	2		294
Chou/Lou [24]	2011	15	15	20	69	Further	P	Net	24	14	3		275
Dennerlein [27]	2007	6	4			Close	C	Net	4				
Donkers [28]	1993	9	9	20–30	78	Further	P	Net	23	12	3		304
Emmatty [41]	2021	30	30	24	67	Further	W	Net	1				
Essers [29]	2013	3	3	31	76	Further	R	Net	3				
Finsen [30]	1997	8	0	46	63	Close	SC	Net	3			2	
Gottlieb [31]	1996	8				Further	R	Net	9				
									13			0.9	
									20			2.2	
									22			3.1	
Hong [32]	1994	6	6			Further	R	Net	10				
									14			0.9	
									20			2.2	
									25			3.1	
Hussain [33]	2020	1	0		66	Close	SC	Net	1		1		
King [9]	2019	1	1			Further	C	Net	12		10	2.3	450
Lou [34]	2001	10	10	26	69	Furter	P	Net	22	10	9		
Murray [35]	2004	10	10	34		Close	SC	Net	6	1	0.03		
Okunribido [36]		8	8	26	74	Close	W	Net	76		34	37	
Ratzlaf [37]	2019	10	6		66	Close	SC	Net	2	1			
Sainburg [38]		13	5	28–46		Further	R	Interaction	25			1.2	
								Net	19				
Topka [39]		10		47		Further	R	Interaction	7				
Yamasaki [40]		9	5	22		Close	C	Interaction	10			0,5	
	Total	256	Mean	30	69				17	9	8		337
			St.Dev	10	5				15	5	9		55

*C* cyclic task, *P* push-up task, *R* reaching task, *SC* selfcare task, *W* work

#### Type of ADL task

As ADL tasks are heterogeneous, it was decided to divide them into categories: 1) close to the body and 2) further away from the body: tasks are classified as further away if the position of the shoulder was > 90 anteflexion and/or > 45 abduction. Tasks were then subdivided into: 1) cyclic flexion/extension, 2) push-up, 3) reaching, 4) self-care, 5) work. The subdivisions were chosen based on the aim of the task (selfcare, push-up, work) or a specific type of movement (reaching, cyclic flexion–extension movement). Some articles tested several conditions with external weight. In those cases, the condition with the lowest external weight applied was taken for further analysis; other conditions are reported in Table 1.

#### Elbow joint load

Nineteen studies [9, 15–17, 19, 21–26, 28, 29, 31, 32, 34, 38, 39, 41] reported on tasks that are classified as further away, nine [18, 20, 27, 30, 33, 35–37, 40] reported on tasks close to the body. These studies were then further classified into six studies on cyclic flexion–extension tasks [9, 15, 18, 20, 27, 40], nine on push-up tasks [16, 21–26, 28, 34], seven on reaching tasks (i.e. reaching, pointing) [17, 19, 29, 31, 32, 38, 39], four on self-care tasks (i.e. dentistry, eating, drinking, brush head) [30, 33, 35, 37] and two on a work task (i.e. heavy: pushing trolley, light: sorting waste) [36, 41] (Table 1). Twenty-five studies reported net joint torque [9, 15–37, 41], two studies reported interaction torque [39, 40] and one study reported both interaction and net torque [38]. In addition, nine studies that reported net joint torque (step 3, Fig. 1), also reported bone-on-bone contact force (step 4, Fig. 1) [16, 21–26, 28].

**Fig. 3:**
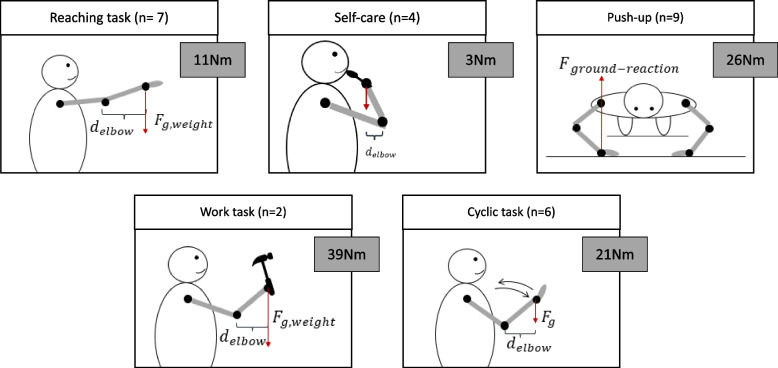
Average net joint moment (flexion–extension), classified per type of task

Twenty-five articles [9, 15–22, 24–38, 41] reported on elbow flexion–extension net joint torque (Table 1). Mean elbow flexion–extension net joint torque was 18 ± 26 Nm for tasks close to the body and 19 ± 13Nm for tasks further away from the body. More specifically, 21 ± 18 Nm for cyclic tasks, 26 ± 11 Nm for pushup tasks, 11 ± 6 Nm for reaching tasks, 3 ± 2 Nm for self-care tasks, and 39 ± 53 Nm for work tasks (Fig. 3). Three articles [38–40] reported on elbow flexion–extension interaction torque, with 10 Nm for mean elbow flexion–extension torque in tasks close to the body and 16 ± 13Nm for the further away tasks.

Ten articles [17, 21, 22, 24–26, 28, 34, 35, 37] reported on varus-valgus net joint torque (Table 1). Mean varus-valgus net joint torque was 1 Nm for tasks close to the body and 11 ± 5 Nm for tasks further away from the body. More specifically, 1 Nm for selfcare tasks, 3 Nm for reaching tasks and 12 ± 5 Nm for pushup tasks.

Eleven articles [9, 17, 21, 22, 24, 26, 28, 33–36] reported on pronation-supination net joint torque (Table 1). Mean pronation-supination net joint torque was 18 ± 19 Nm for tasks close to the body and 6 ± 6 Nm for tasks further away from the body. More specifically, 1 Nm for selfcare and reaching tasks, 7 ± 6 Nm for pushup tasks, 10 Nm for cyclic tasks, and 34 Nm for work tasks.

Nine studies [9, 16, 21–26, 28] reported bone-on-bone contact force. Eight [16, 21–26, 28] of them were pushup tasks, with a reported 337 ± 62 N mean bone-on-bone contact force. One study [9], a cyclic flexion extension task, reported 450 N bone-on-bone contact force.

## Discussion

Aim of the current review was to scope the literature on the reported elbow joint loads during ADL. To this end, in the following section these loads will be compared with published data from retrieval and finite element studies to see if values exceed the failure limits of the prosthesis. The most important finding of this review is that very little literature on elbow joint loading during ADL is available and that our current postoperative instruction does not appear to be evidence-based.

When comparing tasks close to the body with tasks further away from the body, those further away expectedly result in higher loads, as the longer the moment arm of the contributing muscles, the bigger the moment. Our review confirms this. It therefore seems safer to perform ADL tasks that are close to the body or perform tasks in such a way that the distance away from the body is minimized (elbow flexion and shoulder adduction). The highest elbow flexion–extension net joint load for tasks further away from the body was 19 Nm. In addition, work and push-up tasks resulted in the highest flexion–extension loads (39 Nm and 26 Nm, respectively). Especially heavy work (pushing a 37 kg trolley) resulted in high loads (76 Nm). As there is no literature available reporting on failure limits of load on the prosthetic materials for FE movements, whether failure limits would be exceeded at those moments and what the clinical implications are both remain unknown. Both work and push-up tasks result in loads that surpass our post-operative instruction, to not exceed 1 kg regularly and only 5 kg incidentally. Self-care tasks (i.e. dentistry, eating, drinking, brush head), cyclic movements and reaching results in loads that remain below our post-operative instruction.

Highest varus-valgus loads were reported for tasks further away from the body (11 Nm)—more specifically, the highest loads were reported for the push-up tasks (12 Nm). It is known from finite element studies that a varus-valgus load of 5 Nm can lead to irreversible PE deformation [8]. Comparison of our results to available literature shows that all push-up tasks, as well as hammering with a 2 kg hammer in the hand, resulted in moments that led to stresses exceeding the limit of irreversible plastic deformation. These activities thus need to be avoided following TEA. Similar results are reported by King et al. [9], where cyclic flexion–extension with 2.3 kg weight in the hand resulted in a moment in the elbow that led to stresses exceeding the yield strength of PE. This was the case for the condition with 45- and 90-degree shoulder abduction. The condition with 0 degrees shoulder abduction did not exceed the yield strength of PE. It is therefore important to not only report on the movements or tasks being executed and the amount of external weight applied, but also on the distance of the elbow joint in relation to the body (i.e. shoulder position), since similar movements with similar weights can lead to different loads depending on how the movement is executed.

Highest pronation-supination loads were reported in tasks close to the body (18 Nm), more specifically in work tasks (i.e. pushing a 37 kg trolley) (34 Nm). The mean pronation-supination (PS) loads were lower than flexion–extension loads, as can be expected due to shorter moment arms of contributing muscles. As there is no literature available reporting on failure limits of load on the prosthetic materials for PS movements, whether failure limits would be exceeded at those moments and what the clinical implications are both remain unknown.

Highest bone-on-bone contact forces are reported for a cyclic flexion–extension task while holding a 2.3 kg weight in the hand (450 N). Bone-on-bone contact forces during push-up tasks range from 275 to 441 N (mean 337 N). Unfortunately, none of the articles reported between which bones the bone-on-bone contact force was calculated. Finite element analyses evaluating three different prosthetic designs (hourglass, concave and cylindrical) showed that by applying a 100 N axial load, the stresses of both the hourglass and concave designs remained far below (< 50%) the yield strength of PE [42]. The cylindrical design, by contrast, showed the highest stress under these loads, with stresses exceeding the yield strength of PE. The amount of applied load that would result in the PE yield strength being exceeded in both the hourglass and concave designs, was not specified, so clinical implications for these types of prostheses remain unknown. So far, it is known that implant design, type of load, type of movement, frequency of movement cycles, and fixation methods influence the stress distribution on the prosthesis, thereby affecting the risk of prosthetic loosening [43–45]. The consequences of these findings for daily practice remain unclear.

### Recommendations for future research

The results of our review provide a very narrow initial overview of elbow joint loads during ADL, given the limited availability of literature on this topic. It is shown that elbow joints loads (both varus-valgus moment and bone-on-bone contact force) in several ADL tasks exceed the reported failure limits of elbow prostheses. Besides, elbow joint loads also surpass our current post-operative instruction. However, current literature is not sufficient to formulate a new post-operative instruction, which is compliant with failure limits of the prosthesis. We therefore formulate two recommendations for future research, that should be addressed.

First, clinical studies should focus on a thorough analysis of different ADL tasks, since several relevant conditions (i.e. cycling, driving a car, opening a door, carrying groceries) are not yet tested. We advise using a standard set of ADL tasks, which should comprise at least one personal care task, feeding task, housework task, and transportation task [46]. These clinical studies should be done in both healthy participants and patients following TEA, so differences can be analyzed following surgery.

Second, all prosthetic suppliers should test their prosthesis and report failure limits, since different types of prostheses may have different failure limits [42]. We advise to report flexion–extension moment, varus-valgus moment and pronation-supination moment, as well as bone-on-bone contact forces (e.g. axial compression forces) for both clinical and prosthetic studies. Additionally, we advise using net joint torque definitions and calculations and bone-on-bone contact force definitions and calculations, as described in our Methods section so results can be compared [47–49]. This will enable clinicians to compare clinical loading with reported failure limits of the prosthesis and thereby guide informed decisions on post-operative instructions for patients, aiming to improve survival rates.

Last, formulating postoperative instructions might be difficult, since translating a mass into a joint moment is difficult. As mentioned previously, depending on the type of movement and how it is executed, similar weights can lead to different loads on the elbow. The focus should therefore lie more on a balance in load and load capacity and on the execution of the movement (i.e. close to the body, elbow flexion and shoulder adduction vs further away, elbow extension, shoulder abduction), instead of the amount of mass being lifted as is current practice.

### Limitations

The results of this review should be interpreted in light of several limitations caused by the quality of the included articles. Three studies were of low quality, 22 of moderate quality, and three of high quality. In addition, many studies used different measurement systems and methods to calculate the joint load, frequently without reporting validity and reliability, as presented in the quality assessment. Further, different definitions of joint load are reported, making comparison of loads is difficult. Last, the included studies mostly measured young healthy males, which may not be comparable to joint-loading in patients following TEA.

## Conclusion

The results of our scoping review provide an initial overview of the current knowledge on elbow joint loads during ADL. Surprisingly, the current literature is not sufficient to formulate a postoperative instruction for elbow joint loading, which is compliant with failure limits of the prosthesis. Plus, our current instruction does not appear to be evidence-based. Our recommendations, as described previously, offer a starting point in order to assist clinicians in providing informed decisions on post-operative instructions for their patients.

## Supplementary Information

**Additional file 1.**

## Data Availability

The datasets used and/or analyzed are available from the corresponding author upon reasonable request.

## Abbreviations

TEA: Total elbow arthroplasty
PE: Polyethylene
ADL: Activities of daily living
IMU: Inertial measurement unit
FE: Flexion–extension
PS: Pronation-supination
