# Supplementary material for: Elbow joint biomechanics during ADL focusing on total elbow arthroplasty - a scoping review
Source: BMC Musculoskelet Disord. 2023 Jan 18;24:42. doi: 10.1186/s12891-023-06149-8 (PMC9847152; doi:10.1186/s12891-023-06149-8)
Supplement: Supplementary file 1 — Additional file 1. [file 12891_2023_6149_MOESM1_ESM.docx]

**Appendix 1**

("Elbow"[Mesh] OR elbow*[tiab] OR "Elbow Joint"[Mesh] OR "Arthroplasty, Replacement, Elbow"[Mesh] OR TEA[tiab])

**AND**

("Biomechanical Phenomena"[Mesh] OR biomechanic*[tiab] OR kinematic*[tiab] OR kinetic*[tiab] OR (load*[tiab] AND (joint*[tiab] OR distribution*[tiab] OR transmission*[tiab] OR transmit*[tiab] OR carriage*[tiab])) OR mechanical force*[tiab])

**AND**

(flexion[tiab] OR extension*[tiab] OR varus[tiab] OR valgus[tiab] OR pronation[tiab] OR supination[tiab])

NOT ("Sports"[Mesh] OR "Cerebral Palsy"[Mesh] OR "Stroke"[Mesh] OR "Down Syndrome"[Mesh] OR "Spinal Cord Injuries"[Mesh]) NOT ("animals"[MeSH] NOT "humans"[MeSH])

TS= (elbow* OR tea)

AND

TS= (biomechanic* OR kinematic* OR kinetic*[tiab] OR (load* AND (joint* OR distribution* OR transmission* OR transmit* OR carriage*)) OR “mechanical force*”)

AND

TS=(flexion OR extension* OR varus OR valgus OR pronation OR supination) NOT TI=(sport* OR “cerebral palsy” OR “down syndrome” OR “spinal cord injuries” OR stroke)

**Appendix 2**

Quality assessment criteria checklist

| **Questions** | **Response / decision rule criteria** |
| --- | --- |
| 1. Participant characteristics – are participant demographics adequately described? (include: number, age, gender) | Adequate – all details provided  Partial – all details except level of competition or gender  Inadequate – missing details |
| 2. Were inclusion/exclusion criteria stated? | Stated - clear list of both given  Limited - one or two points only  Not stated - not provided |
| 3. Was the design appropriate to the research question? | Yes - well matched to question  No |
| 4. Were key-dependent variables measured? | Adequate - all details provided  Partial - only some variables measured  Inadequate - missing details |
| 5. Psychometric properties - was the reliability of measurement tools reported and adequate? | Adequate - all details provided  Partial - only some aspects of reliability reported  Inadequate - missing details |
| 6. Psychometric properties —was the validity of measurement tools reported and adequate? | Adequate - all details provided  Partial - only some aspects of validity reported  Inadequate - missing details |
| 7. Was the external validity of the results discussed? | Yes - generalizability of findings discussed  No |
| 8. Were the limitations of the studies described? | Adequate - all limitations discussed  Partial - limited description  Inadequate - not described |

**Appendix 3**

| Study First Author | Year | Are participant demographics adequately described? (include: number, age, gender) | Were inclusion/exclusion criteria stated? | Was the design appropriate to the research question? | Were key-dependent variables measured? | Psychometric properties - was the reliability of measurement tools reported and adequate? | Psychometric properties —was the validity of measurement tools reported and adequate? | Was the external validity of the results discussed? | Were the limitations of the studies described? | Total (max 8) | Category |
| --- | --- | --- | --- | --- | --- | --- | --- | --- | --- | --- | --- |
| Almeida (15) | 1995 | 0,5 | 0 | 1 | 1 | 0,5 | 0 | 1 | 0 | 4 | M |
| An (16) | 1992 | 0 | 0 | 1 | 0 | 0 | 0 | 0 | 0 | 1 | L |
| Balendra (17) | 2017 | 1 | 1 | 1 | 0,5 | 1 | 0 | 1 | 1 | 6,5 | H |
| Ballaz (18) | 2016 | 0,5 | 0,5 | 1 | 0,5 | 1 | 0 | 1 | 1 | 5,5 | M |
| Beer (19) | 2004 | 0,5 | 0 | 1 | 1 | 0 | 0,5 | 1 | 0,5 | 4,5 | M |
| Challis (20) | 1994 | 0,5 | 0 | 1 | 0 | 0 | 0,5 | 1 | 1 | 4 | M |
| Chou (21) | 2001 | 1 | 0 | 1 | 0,5 | 0 | 0,5 | 1 | 0 | 4 | M |
| Chou (22) | 2002 | 1 | 0 | 1 | 1 | 0 | 0,5 | 1 | 0,5 | 5 | M |
| Chou (25) | 2008 | 0,5 | 0 | 1 | 0,5 | 0 | 0,5 | 1 | 1 | 4,5 | M |
| Chou (23) | 2009 | 1 | 0 | 1 | 0 | 0 | 0,5 | 1 | 1 | 4,5 | M |
| Chou/Hsu (26) | 2011 | 1 | 0,5 | 1 | 1 | 0 | 0,5 | 1 | 0 | 5 | M |
| Chou/Lou (24) | 2011 | 1 | 0,5 | 1 | 1 | 0 | 0,5 | 1 | 0 | 5 | M |
| Dennerlein (27) | 2007 | 0,5 | 0 | 1 | 1 | 0 | 0,5 | 1 | 1 | 5 | M |
| Donkers (28) | 1993 | 1 | 0,5 | 1 | 1 | 0 | 0,5 | 1 | 1 | 6 | H |
| Emmatty (41) | 2021 | 1 | 0.5 | 1 | 0.5 | 1 | 1 | 0 | 0.5 | 5,5 | M |
| Essers (29) | 2013 | 1 | 0,5 | 1 | 0,5 | 0 | 0 | 1 | 1 | 5 | M |
| Finsen (30) | 1997 | 1 | 0 | 1 | 0 | 0 | 0 | 1 | 0 | 3 | L |
| Gottlieb (31) | 1996 | 0 | 0 | 0,5 | 1 | 0 | 0,5 | 1 | 1 | 4 | M |
| Hong (32) | 1994 | 0 | 0 | 1 | 0,5 | 0 | 0,5 | 1 | 0 | 3 | L |
| Hussain (33) | 2020 | 0,5 | 0 | 1 | 0,5 | 1 | 1 | 1 | 0,5 | 5,5 | M |
| King (10) | 2019 | 0,5 | 0 | 1 | 1 | 0,5 | 0,5 | 1 | 1 | 5,5 | M |
| Lou (34) | 2001 | 1 | 0 | 1 | 0,5 | 0,5 | 0,5 | 1 | 0 | 4,5 | M |
| Murray (35) | 2004 | 0.5 | 0 | 1 | 0.5 | 1 | 0,5 | 1 | 1 | 4,5 | M |
| Okunribido (36) |  | 1 | 0 | 1 | 0,5 | 0,5 | 0,5 | 1 | 1 | 5,5 | M |
| Ratzlaf (37) | 2019 | 0,5 | 0,5 | 1 | 0,5 | 0 | 0,5 | 1 | 1 | 5 | M |
| Sainburg (38) |  | 0,5 | 0 | 1 | 0,5 | 0 | 0,5 | 1 | 1 | 4,5 | M |
| Topka (39) |  | 0,5 | 0 | 1 | 1 | 0,5 | 0,5 | 1 | 1 | 5,5 | M |
| Yamasaki (40) |  | 0,5 | 0 | 1 | 1 | 1 | 1 | 1 | 1 | 6,5 | H |
|  |  |  |  |  |  |  |  |  | Mean | 4,7 |  |

Overview of quality assessment. L = low quality, M = moderate quality, H = high quality.
